# Physiologic Stresses Reveal a *Salmonella* Persister State and TA Family Toxins Modulate Tolerance to These Stresses

**DOI:** 10.1371/journal.pone.0141343

**Published:** 2015-12-03

**Authors:** Eugenia Silva-Herzog, Erin M. McDonald, Amy L. Crooks, Corrella S. Detweiler

**Affiliations:** Department of Molecular, Cellular and Developmental Biology, University of Colorado, Boulder, Colorado, United States of America; University of Manchester, UNITED KINGDOM

## Abstract

Bacterial persister cells are considered a basis for chronic infections and relapse caused by bacterial pathogens. Persisters are phenotypic variants characterized by low metabolic activity and slow or no replication. This low metabolic state increases pathogen tolerance to antibiotics and host immune defenses that target actively growing cells. In this study we demonstrate that within a population of *Salmonella enterica* serotype Typhimurium, a small percentage of bacteria are reversibly tolerant to specific stressors that mimic the macrophage host environment. Numerous studies show that Toxin-Antitoxin (TA) systems contribute to persister states, based on toxin inhibition of bacterial metabolism or growth. To identify toxins that may promote a persister state in response to host-associated stressors, we analyzed the six TA loci specific to *S*. *enterica* serotypes that cause systemic infection in mammals, including five RelBE family members and one VapBC member. Deletion of TA loci increased or decreased tolerance depending on the stress conditions. Similarly, exogenous expression of toxins had mixed effects on bacterial survival in response to stress. In macrophages, *S*. Typhimurium induced expression of three of the toxins examined. These observations indicate that distinct toxin family members have protective capabilities for specific stressors but also suggest that TA loci have both positive and negative effects on tolerance.

## Introduction

Persister bacteria play an important role in recalcitrant chronic infections and their hallmark is non-heritable stress tolerance [1,2]. Joseph Bigger was the first to note that a culture of growing bacteria cannot be completely killed even with high levels of antibiotic, and he called the tolerant survivors ‘persisters’ [3]. Persister populations have since been identified upon treatment of bacteria with multiple classes of antibiotics and under broad environmental stress conditions [4–6]. In the laboratory, persisters are identified with a biphasic curve as the surviving cell population after an initial rapid kill of the majority of the bacteria within a population. In other words, persisters are the cell population that survives exposure to bactericidal stress despite being genetically susceptible [7,8]. However, whether persisters arise in response to stress or pre-exist within a phenotypically heterogeneous population of isogenic bacteria or both remains a matter of debate [9,10]. Regardless, these cells play important roles in chronic infections and tolerance to antibiotic therapy. Thus, persisters may represent a survival strategy for a clonal population, enabling some individuals to withstand and later recover from environmental stress, such as those encountered within a host.

Toxins from Toxin-Antitoxin (TA) loci promote a persister state and thereby contribute to tolerance of environmental challenges [11]. Toxins promote tolerance by directly or indirectly inhibiting growth, for instance by blocking essential cellular processes, such as DNA replication, or by causing damage that leads to arrest [12–15]. Antitoxins restore cellular activity by inhibiting their cognate toxin, which, in turn, restores sensitivity to stress [16]. Among the different classes of TA systems, type II loci encode protein antitoxins that inhibit toxins by direct protein-protein contact. Type II TA loci are more abundant in free-living bacteria and archaea as compared to obligate intracellular organisms [17]. Type II TAs are also more abundant in 12 genomes representing "the most dangerous pandemic bacteria" compared to their closest nonpathogenic or non-epidemic counterparts [18]. For instance, pathogenic *Mycobacterium tuberculosis* H37Rv has 62 TA Type II loci, whereas nonpathogenic *M*. *smegmatis* has four. *Salmonella enterica* serovar Typhimurium causes systemic and enteric infections in a wide variety of hosts and encodes 19 TA loci [19], whereas *S*. *enterica* Typhi causes infections specifically in humans and chimps and only has 12 TA loci. Finally, *S*. *enterica* arizonae causes gastroenteritis and has only eight TA loci, and nonpathogenic *S*. *bongori* has three [20]. These observations suggest that pathogenic strains accumulate type II TA systems, perhaps to provide tolerance to diverse conditions encountered within microenvironments in the host.

In mice, *S*. *enterica* Typhimurium causes murine typhoid fever, a systemic infection that models both the acute and chronic stages of human typhoid fever. Ingested bacteria cross the intestinal epithelium and reside inside professional phagocytes, particularly macrophages located in the liver, spleen, and lymph nodes [21,22, 23]. Recent studies have shown that TA systems are required for *S*. Typhimurium survival in the host and for tolerance to antibiotics [19,24–26]. For instance, the *sehAB* TA system is required in the mesenteric lymph nodes, and to a lesser extent in spleen and liver [24]. In addition, it was recently demonstrated that the toxins *vapC2* and T4 are needed for survival in a fibroblast cell line, whereas in HeLa cells only *vapC2* is required [19]. Thus, some TA systems are dispensable in particular host cell types, suggesting that the microenvironment plays an important role in determining which TAs are needed for bacterial survival [19,27,28].

One way to establish whether toxins or sets of toxins may be important for tolerance under specific conditions is to ectopically express toxins and monitor tolerance to stress. In *S*. Typhimurium, a mutant strain (shpAB*) that co-expresses the ShpB toxin with a truncated, unstable ShpA antitoxin has an increased percentage of cells tolerant to antibiotics [26]. The ectopic expression of the *E*.*coli* RelE toxin in broth not only arrests growth but also increases tolerance to several classes of antibiotics [29]. Whether the tolerance provided by ectopic expression of these individual toxins extends to other stresses is not known.

In this study, we first demonstrate the reversibility of *S*. Typhimurium tolerance to antibiotics and to conditions that mimic stresses present within the macrophage phagosome, indicating that surviving bacteria represent a persister state. *S*. Typhimurium has at least 19 type II TA systems [19,24,25]. We address whether the six chromosomal TA systems conserved across *Salmonella enterica* serovars that colonize mammals and birds may increase tolerance to physiological stress. The data show that different RelBE family members modulate tolerance in response to distinct stress conditions.

## Materials and Methods

All animal work was conducted according to national and international guidelines and with the approval of the Institutional Animal Care and Use Committee of the University of Colorado Boulder, protocol number 1307.02.

### Bacterial strains and growth conditions

*S*. Typhimurium strain SL1344 [30] was used for these studies. Bacterial strains and plasmids are described (Table 1). Bacteria were grown on Luria-Bertani (LB) agar plates or in LB broth. M9 minimal media supplemented with 0.002% histidine and 2% glucose or 0.4% glycerol were used as noted. Cells were grown by dilution of overnight cultures 1:100 in 12 to 25 mL of media and incubated in 125 mL culture flasks at 37°C with aeration. Antibiotics (Sigma-Aldrich; St Louis, MO) were used at the following concentrations: streptomycin at 200 μg/mL, chloramphenicol at 40 μg/mL and ampicillin 50 μg/mL. Colony heterogeneity was monitored upon plating to LB-agar supplemented with 0.2% arabinose (Sigma-Aldrich) in the presence of appropriate antibiotics. Growth curves and growth arrest were monitored by OD_600_ in a microtiter plate reader (BioTeck, Winooski, VT).

**Table 1:** Bacterial strains and plasmids.

Strain or plasmid	Relevant Genotype	Reference
***Strains***		
**TOP10**	*E*. *coli* F- *mcrA* Δ(*mrr-hsdRMS*-*mcrBC*) φ80l*acZ*ΔM15 Δl*ac*X74 *nupG recA*1 *ara*D139 Δ(*ara-leu*)7697 *galE*15 *galK*16 *rpsL*(Str^R^) *endA*1 λ^-^	Invitrogen
**SL1344**	*S*.Typhimurium *hisG xyL rpsL (*wild-type)	Smith et al 1984
**Sl1344 Δ2TA**	Δ*relBE* Δ*relBE-3*	This study
**Sl1344 Δ3TA**	Δ*relBE* Δ*relBE-3* Δ*sehAB*	This study
**Sl1344 Δ4TA**	Δ*relBE* Δ*relBE-3* Δ*sehAB* Δ*shpAB*	This study
**Sl1344 Δ5TA**	Δ*relBE* Δ*relBE-3* Δ*sehAB* Δ*shpAB* Δ*relBE-5*	This study
**Sl1344 Δ6TA**	Δ*relBE* Δ*relBE-3* Δ*sehAB* Δ*shpAB* Δ*relBE-5* Δ*vapBC*	This study
***Plasmids***		
pBAD_33_	Cloning vector Cm^r^	Guzman et al
pBAD_24_	Cloning vector Amp^r^	Guzman et al
pBAD_33_-relE	pBAD-*relE*	This study
pBAD_33_-relB	pBAD-*relB*	This study
pBAD_33_-relBE	pBAD-*relBE*	This study
pBAD_33_-vapC	pBAD-*vapC*	This study
pBAD_24_-vapB	pBAD-*vapB*	This study
pBAD_24_-vapBC	pBAD-*vapBC*	This study
pBAD_33_-relE-3^1^	pBAD-*relE-3*	This study
pBAD_33_-relB-3^1^	pBAD-*relB-3*	This study
pBAD_33_-relBE-3^1^	pBAD-*relBE-3*	This study
pBAD_33_-relE-5**^1^**	pBAD-*relE-5*	This study
pBAD_33_-relB-5^**^1^**^	pBAD-*relB-5*	This study
pBAD_33_-relBE-5	pBAD-*relBE-5*	This study
pBAD_33_-sehA**^2^**	pBAD-*sehA*	This study
pBAD_33_-sehB	pBAD-*sehB*	This study
pBAD_33_-sehAB	pBAD-*sehAB*	This study
pBAD_24_-shpAB* **^3^**	pBAD-*shpAB1*	Slattery et al
pBAD_24_-shpAB**^3^**	pBAD-*shpAB*	Slattery et al

1 relE homologues were numbered based on chromosomal order with the exception of relE, which was previously annotated [31].

2 previously identified [24]

3 previously identified [26]

* truncated unstable antitoxin [26].

### Construction of strains with deletions

Strains with deletions of the indicated open reading frames were constructed using a scar-less deletion method [32] with the primers indicated (Table 2).

**Table 2:** DNA primers used in this study.

Name	Sequence
***Cloning primers***	
*relE_Fwd*	CCGGTACCGGATCCAAAATAAGGAGGAAAAAAAAAATGACTTATGAACTGGAATTCGACCC
*relE_Rv*	CCCCCGCATGCGAATTCATGCAGAAATTATAAAGCCGTTTGTTTGCATCG
*relB_Fwd*	GGTACCGGATCCAAAATAAGGAGGAAAAAAAAATGGCCACGCTGAACGTCC
*relB_Rv*	TCCCCAAGCTTATGCAGAAATTATCATAAGTC A TCCAGACTAACCTTG
*relBE-_Rv*	AAGCTTGAATTTTTGCCATGCAGATCATAAAAGCCGTTTG
*vapC_Fwd*	CCCCCGGTACCGGATCCAAAATAAGGAGGAAAAAAAAATGCTGAAATTCATGCTTGATAC
*vapC_Rv*	CCCCCGCATGCGAATTCATGCAGAAATTA
*vapB_Fwd*	CCCCCGGTACCGGATCCAAAATAAGGAGGAAAAAAAAATGCACACAACACTTTTTTTTAG
*vapB_Rv*	CCCCCGCATGCGAATTCATGCAGAAATCAAAATCCTTCCCGTTC
*relE-3_Fwd*	CTGGTACCGGATCCAAAATAAGGAGGAAAAAAAAAATGCGAACCTTCAAAACCAGGTGG
*relE-3_Rv*	GCATGCGGTAAAACCCTCATACCAGCAC
*relBE-3_Fwd*	GAGCTCGGATCCAAAATAAGGAGGAAAAAAAAATGCATGTCAGCAAAAACTAAATTC
*relBE-3_Rv*	GCATGCGGTAAAACCCTCATACCAGCAC
*relE-5_Fwd*	GGTACCGGATCCAAAATAAGGAGGAAAAAAAAATGCGTGGGATCTTCTCTGGAGGATC
*relE-5_Rv*	CTTGCATGCGAATTCAGTTTTGGCAATCACAAGCTG
*relBE-5_Rv*	GCATGCCAATCACAAGCTGTTTCTCCGTTGTTGC
*sehA_Fwd*	GGTACCGGATCCAAAATAAGGAGGAAAAAAAAATGCATGGATGCAACCAGCGC
*sehA_Rv*	GCATGCGACATATCCAACTACTCGATGAAGG
*sehAB_Rv*	CCCCCGCATGCGAATTCATGCATGAGTTCTCATTCTTTAT
shpAB*	[26]
*shpAB*	[26]
***qPCR primers***	
*relE_QFwd*	AAAGTTGGCTGATGTGCTATTG
*relE_QRv*	CAGACGATAACCGGACGATTTA
*vapC_QFwd*	AGAGAACGCTTCAACCTCAATA
*vapC_QRv*	TCCACGACGGCAAGATTAC
*relE-3_QFwd*	GAGTTAAGCGAGGCCATCAA
*relE-3_QRv*	TCCCTTTGCCAACACGATT
*relE-5_QFwd*	AGTATGGAATTGAACCCGATGA
*relE-5_QRv*	GCTTCTTCAAACTTAGCGACATAC
*sehA_QFwd*	CCTGAAGAGATGAAGCGGTATATT
*sehA_QRv*	GGAAATGAGTCTCAGGCTGTTA
*shpA_QFwd*	GTGGTCATTCTGGTTGCTCATA
*shpA_QRv*	CCATGCTCATAACGATTCCTCTC
*16S rRNA_QFwd*	CTGGCGGCAGGCCTAACACA
*16S rRNA_QRv*	CGCCACTCGTCAGCGAAGCA
***Deletion primers***	
*relE*.*st*.*R*	TAAGCTGAGCCTTCACC
*relE*.*st*.*F*	CGTCACTCTTAGCGACAG
*relE-3*.*st*.*F*	CGAGAGTTGAGAATTATCTTCTC
*relE-3*.*st*.*R*	GTCAGTATAGTGCAATTCCC
*relE-5*.*st*.*F*	CCATCACTTGCACCTG
*relE-5*.*st*.*R*	AACGCCGTTTGAACTC
*sehAB*.*st*.*F*	CAACAGGCAAAATGACCT
*sehAB*.*st*.*R*	TTCGAAGGCATTTATATCG
*vapBC*.*st*.*F*	GAGTGCGCGTCATGTG
*vapBC*.*st*.*R*	AATGTGATGCAAACCACC
*shpAB*.*st*.*F*	TTATCTCGAATACACAAATTGTGA
*shpAB*.*st*.*R*	CGGCATTTGTGGGGTTAAG
*relE-5*.*st*.*F*	CCATCACTTGCACCTG
*relE-5*.*st*.*R*	AACGCCGTTTGAACTC

Restriction sites are underlined.

### Plasmid construction

Genes were amplified from SL1344 chromosomal DNA using Q5 high fidelity thermostable polymerase (New England BioLabs; Ipswich, MA), and primers (Integrated DNA Technologies; Coralville, IA) as described in Table 2. PCR fragments were digested with KpnI and SphI, except for *relB*, for which KpnI and HindIII were used (New England BioLabs). Fragments were ligated into the corresponding sites of pBAD_24_ or pBAD_33_ vector as listed on Table 2. Ligation reactions were transformed by electroporation into the *E*. *coli* TOP10 strain (Invitrogen; Carlsbad, CA) and sequenced. Plasmids were transformed into SL1344 by electroporation.

### Determination of Minimal Inhibitory Concentration (MIC)

The MIC of ampicillin, ciprofloxacin and hydrogen peroxide was determined by serial dilution in a 96-well plate with an inoculum of 10^6^ cells. Plates were incubated at 37°C for 22 hrs and then the OD 600 measured. The MIC was determined as the lowest concentration of antibiotic that prevented bacterial growth. The concentration ranges tested follow: ampicillin 6–500 μg/mL; ciprofloxacin 0.005 to 6 μg/mL; hydrogen peroxide 0.007 to 5 mM; polymyxin B 0.05 to 10 μg/mL.

### Tolerance assays

Strains were grown overnight at 37°C in LB broth containing the appropriate antibiotics. A 1:100 dilution of the overnight culture was inoculated into 25 mL M9 minimal media with glucose, and incubated at 37°C with agitation to an OD_600_ of 0.8. Different stresses were added: ampicillin (100 μg/mL; MIC 6 μg/mL for the wild-type and ∆TA6 strains [26]), ciprofloxacin (0.2 μg/mL; MIC 0.8 μg/mL for the wild-type and ∆TA6 strains [13]), hydrogen peroxide (1.25 mM; MIC 0.125 mM for the wild-type and ∆TA6 strains) or dipyridyl (0–25 mM). The hydrogen peroxide was derived from a stock solution stored in single-use aliquots at -80°C. For low pH stress, cells were grown in LB to an OD_600_ of 0.6–0.8, harvested and resuspended in E Minimal Media [33], adjusted to pH 2.6, 3.0, 3.5 or 4.4 and monitored for pH at the end of each experiment. Cultures were returned to 37°C, incubated with agitation for the remainder of the experiment, and sampled over time as indicated. Serial dilutions in PBS were plated to enumerate CFU. A modified microtiter plate assay was used for exposure to polymyxin B (Sigma-Aldrich) [34]. Briefly, overnight cultures were diluted 1:100 in M9-dextrose and grown to an OD of 0.8; cells were diluted to 2.5 x 10^4^ CFU/mL and 100 μL aliquots were incubated in 96-well plates in triplicate with freshly prepared polymyxin B at 5 μg/mL; MIC of 1.25 μg/mL for wild-type strain. Plates were incubated at 37°C with agitation for the designated times and then diluted in PBS to determine CFUs. For tolerance experiments of strains harboring overexpressing plasmids, cultures were grown to an OD_600_ of 0.6–0.8 and induced for 2 hours with 0.2% L-arabinose in M9 supplemented with glycerol before exposure to stress and monitoring by plating for CFU at the times indicated.

### Peroxide quantification

The media from tolerance assays were sampled at 0, 1, 2, 6 and 8 hours after the addition of hydrogen peroxide. Peroxide was quantified using a Pierce Quantitative Peroxide Assay kit (Thermo scientific Rockford, IL) following the manufacturers instructions.

### Persister assays

After 4 hours of exposure to stress in a tolerance assay, surviving bacteria were harvested, washed twice with PBS, resuspended in rich media (LB), and grown at 37°C overnight with agitation. This outgrown culture was then exposed to stress in a tolerance assay as described above. To instead examine individual clones that survived the first 4 hours of exposure to stress, tolerant bacteria were diluted, plated to LB agar and grown overnight at 37°C to obtain discrete colonies. Three individual colonies were inoculated into LB, grown to late log phase at 37°C and then re-exposed to the stress condition.

### RNA isolation and semi-quantitative RT-PCR

Three colonies of wild-type *S*. Typhimurium were grown overnight in LB at 37°C. In triplicate, 50 μL of each overnight culture was transferred to 5 mL of Dulbecco’s Modified Eagle Medium (DMEM). Cultures were then incubated at 37°C for 2 hours. Total RNA was extracted from 0.8 mL of each culture, using the PureLink RNA Mini Kit (Invitrogen), according to the manufacturer’s instructions. RNA concentration was measured by Take3 (BioTek; Winooski, VT). For infections, bone marrow progenitor cells were isolated from the femurs and tibiae of Sv129S6 mice as previously described [35]. The resulting bone marrow-derived macrophages (BMDMs) were pre-treated with Interferon-γ (IFNγ, 20 U/mL) for 18–24 hours, followed by infection with *S*. Typhimurium at a multiplicity of infection of approximately 10, as determined by plating for CFU. The infection proceeded for 2 hours at 37°C in a 5% CO_2_ incubator. The supernatant was then removed and BMDMs were washed twice with 2 mL of 1X PBS (Gibco; Carlsbad, CA). 1 mL of 1x PBS was added to the cells and the BMDMs were scraped up and pipetted directly into a microcentrifuge tube. The cells were centrifuged at 18,000 x g for 10 minutes to lyse the BMDMs and pellet the bacteria. RNA was isolated from the pelleted bacterial cells using the PureLink RNA Mini Kit (Life Technologies; Carlsbad, CA) according to the manufacturer’s instructions. 25–200 ng of RNA was used to prepare cDNA using the Superscript III First-Strand Synthesis Kit (Life Technologies). Primers were designed to amplify a 70–150 base pair region of these toxins: *vapC*, *relE*, *relE-3*, *relE-5*, *parE*, *sehA*, and *shpA* (Table 2). 40 cycles of quantitative reverse transcriptase-polymerase chain reaction (qRT-PCR) were performed using SYBR Green PCR Master Mix (Applied Biosystems; Carlsbad, CA) and 0.2 mM of each primer pair.

### Statistical analyses

Two-way comparisons were analyzed with a Student’s t-test (for parametric data). Multiple comparisons were analyzed by ANOVA with a Tukey's (parametric data) or Dunnett's (nonparametric data, including growth curves) post-hoc test. GraphPad Prism version 6.00 for Mac OS X (GraphPad Software, La Jolla, CA) was used. *P* values < 0.05 were considered significant.

## Results

### A small fraction of *S*. Typhimurium is tolerant to host-associated stressors

Non-replicating bacteria have been observed in primary macrophages and in mice infected with *S*. Typhimurium, suggesting a persister state [25]. To establish whether exposure to conditions that partially mimic stresses found in the macrophage vesicle reveal a tolerant population, we monitored changes in the number of *S*. Typhimurium that survive exposure to oxygen radicals, low pH, cationic antimicrobial peptides, or iron-limiting concentrations. The concentration of each stress used for these experiments, was selected based on 1% or less bacterial survival [13,26] and/or known physiological concentrations in the macrophage phagosome. For example, in the Salmonella Containing Vacuole (SCV), the pH declines to less than 4.5 within eight hours of infection [36], and hydrogen peroxide can accumulate to concentrations in the millimolar range [37]. While polymyxin B is not produced by mammals, other cationic antimicrobial peptides are within the macrophage SCV, but their concentrations have not been established [38]. The iron concentration in the *S*. Typhimurium-containing phagosome is also unknown, but treating macrophages with the ferrous iron chelator dipyridyl (0.2 mM) limits *S*. Typhimurium replication in broth and in macrophages [39–41].

**Fig 1:**
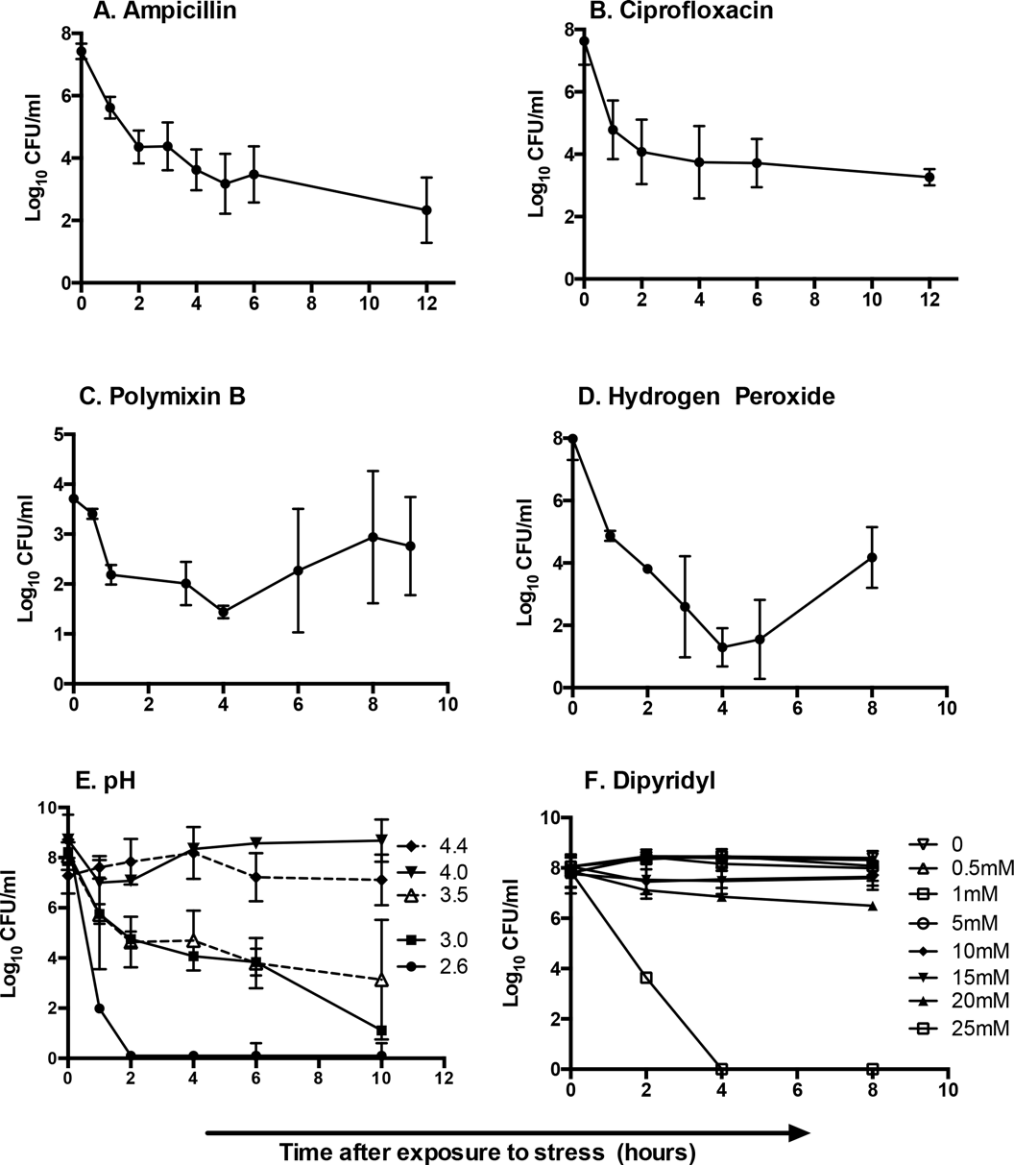
A small fraction of *S*. Typhimurium is tolerant to conditions that mimic stressors associated with infection. Bacteria were grown to late log phase in LB and exposed to antibiotics (A) ampicillin, 100 μg/mL, (B) ciprofloxacin, 0.2 μg/mL, (C) polymyxin B, 5 μg/mL, (D) hydrogen peroxide, 1.25 mM, (E) pH 2.6–4.4, or (F) dipyridyl, 0–30 mM. Bacteria were monitored thereafter for survival by plating to LB-agar plates and enumerating CFU. Mean of > 4 independent experiments are shown; error bars denote SEM.

Within two hours of exposure to antibiotics, hydrogen peroxide or polymyxin B, a small fraction of bacteria (1.0–0.1%) was recovered upon plating, revealing the portion of the population tolerant to the corresponding stress (Fig 1). Over the course of the incubation with hydrogen peroxide, peroxide declined 20% within 2 hours but remained five-fold higher than the MIC. By eight hours, peroxide declined more than 90%, dropping below the MIC. In the analyses of pH, most (>90%) bacteria survived pH 4.4. At pH 4.0 or 3.5, approximately 15% of bacteria survived after two hours, but only 0.1% were tolerant to pH 3.0. Essentially no bacteria recovered after one hour at pH 2.6. Exposure to the iron chelator dipyridyl did not reveal a tolerant population at any concentration tested, and the highest concentration was fully lethal. In summary, exposure of *S*. Typhimurium to oxidative stress, pH 3.5 or 3.0, or cationic antimicrobial peptide uncovers a subpopulation of tolerant bacteria.

### *S*. Typhimurium cells that are tolerant to hydrogen peroxide or low pH have adopted a persister state

**Fig 2:**
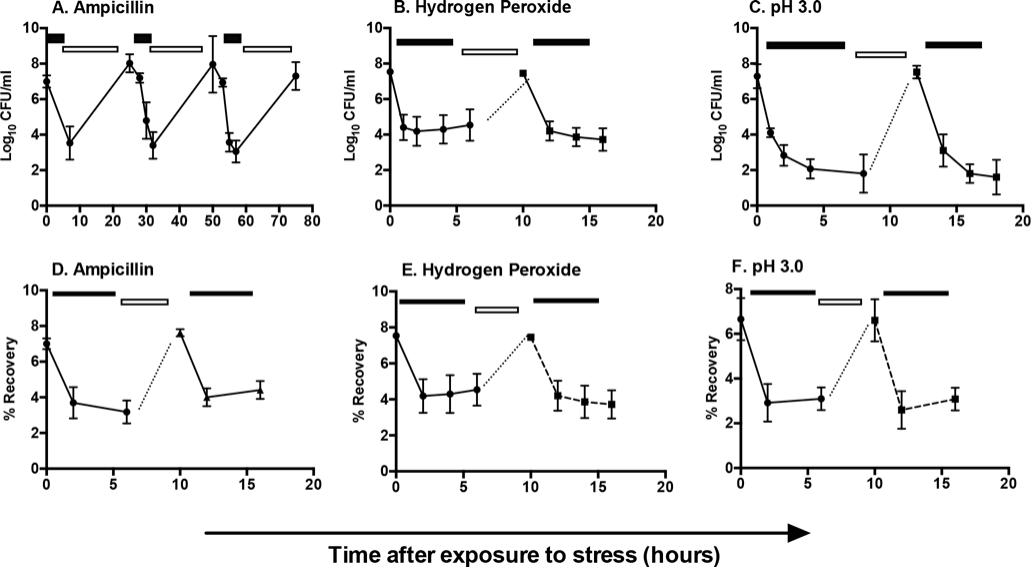
*S*. Typhimurium tolerance represents a persister state characterized by phenotypic variation. Bacterial CFU were monitored after multiple rounds of treatment with (A) ampicillin, 100 μg/mL, (B) hydrogen peroxide, 1.25 mM, or (C) pH 3.0. Presence of stress is indicated by filled rectangles and regrowth without stress by open rectangles. Alternatively, (D-F) independent colonies were grown to late log phase (dotted line) and challenged with the corresponding stressors as indicated by the filled rectangles. Mean of > 3 independent experiments are shown; error bars denote SEM.

To determine whether *S*. Typhimurium tolerance to antibiotics or other stressors represents a transient, reversible state characteristic of persisters, we grew-type *S*. Typhimurium to log phase and then exposed cultures to antibiotic, hydrogen peroxide or pH 3.0 for four hours. Surviving bacteria were recovered in LB for six to eight hours and then re-exposed to the stress. Alternatively, after four hours of stress, surviving bacteria were plated to LB agar and grown overnight. Three individual colonies were inoculated into LB, grown to late-log phase, and exposed to stress for a second time. Regardless of whether tolerant bacteria were recovered as a population (in broth, Fig 2A–2C) or as individual clones (colonies, Fig 2D–2F), we observed a subsequent steep decline in the percentage of tolerant cells upon re-exposure to stress. These data are consistent with a transient persister state, not genetically resistant mutants, and suggest that persisters are not limited to antibiotic tolerance. In addition, the tolerant population appears to consist not of a single clone that becomes dominant upon outgrowth, but of multiple individual persisters that recover when environmental conditions improve.

### RelBE and VapBC TA Systems modulate tolerance in *S*. Typhimurium

The genus *Salmonella* comprises two species, *S*. *enterica* and *S*. *bongori*. *S*. *enterica* includes serovars that cause systemic, chronic disease in mammals and birds, whereas *S*. *bongori* colonizes the gastrointestinal tract of reptiles, often asymptomatically [42]. To identify TAs that may promote *S*. Typhimurium tolerance to host-associated conditions that select for persisters, we focused on *S*. Typhimurium chromosomal TA systems that represent a known TA superfamily, are missing from *S*. *bongori*, and are present in at least four out of six *S*. *enterica* species examined (Table 3, [24]). Of these six TA loci, one encodes VapBC and five encode RelBE family members. Two of the RelE family members, ShpA and SehA are demonstrated toxins, and ShpAB was previously identified in a screen for *S*. Typhimurium mutants with increased tolerance to antibiotics [24,26].

**Table 3:** S. Typhimurium TA System conservation^a^.

	Locus Tags for *S*. Typhimurium strains^b^		Name	TA Superfamily	*S*. *bongori*	*S*. *enterica*					
						*arizonae* ^*c*^	*enterica*				
							Paratyphi			Typhi	
#	SL1344	LT2					A	B	C	CT18	Ty2
**1**	1479–1480	1550–1551	RelBE-2	RelBE	0	1	1^d^	1^d^	1^d^	1^d^	1^d^
**2**	2884–2885	2904–2905	*ta2*	*Unclassified*	0	0	1	1	1	1	1
**3** ^**f**^	2935-2936^e^	2954–2955	ParDE		0	0	1^e^	0	0	1^e^	1^e^
**4** ^f^ ^,^ ^h^	3011–3012	3033–3034	VapBC	VapBC	0	0	1	1	1	1	1
**5**	3437–3448	3470–3471	*ta3*	*Unclassified*	1	1	1	1	1	1	1
**6**	3483–3484	3516–3517	DinJ-YafQ	RelBE	0	0	0	0	0	0	0
**7**	3617–3618	3652–3651	*ta4*	*Unclassified*	1	1	1	1	1	1	1
**8** ^f^ ^,^ ^h^	3743–3744	3777–3778	RelBE-3	RelBE	0	0	1^e^	1	1	1	1
**9** ^h^	3866–3867	3906–3907	RelBE-5	RelBE /HigBA	0	0	1	1	1	1	1
**10** ^f^ ^,^ ^h^	3976-3977^f^	4030–4031	SehAB	RelBE	0	0	1	1	0	1	1
**11** ^f^	3979-3980^f^	4032–4033	SehCD	RelBE	0	1	0	0	1	0	0
**12**	4253–4254	4317–4318	*ta5*	*Unclassified*	1^e^	1^e^	1	1^e^	1^e^	1	1
**13** ^f^ ^,^ ^h^	4379–4380	4449 4450	RelBE	RelE	0	1	1	1	1	1	1
**14** ^g^ ^,^ ^h^	4459-4460^g^	4528–4529	ShpAB	RelBE	0	0	1	1	1	1	0

a Conservation was established with the Integrated Microbial Genomes on-line tool [43].

b Genes are present in strains UK1 and 14028s as well as SL1344 and LT2; toxins are underlined and listed first.

c S. enterica arizonae colonizes but is poorly able to cause systemic infection in mammals [44]

d The toxin is conserved as indicated, but an adjacent putative antitoxin is not present [20].

e The antitoxin is conserved as indicated, but an adjacent putative toxin is not present [20].

f Previously identified as absent from S. bongori and conserved across S. Typhimurium, Typhi and Paratyphi B [24].

g Identified in a screen for transposon insertions that increase tolerance to antibiotics [26].

h Identifies loci examined in this study.

**Fig 3:**
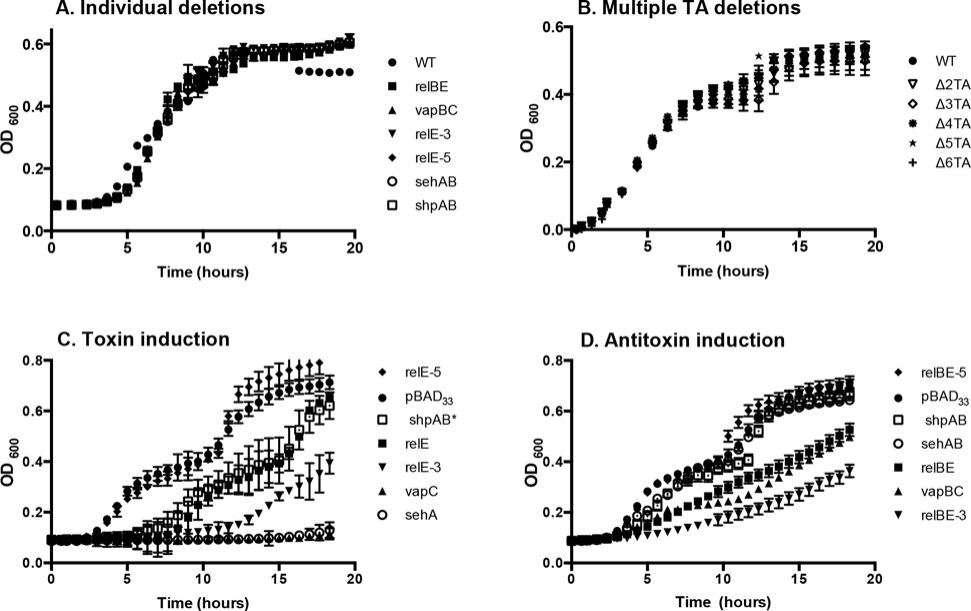
Expression of toxins arrests or delays growth upon induction, but none of the toxins is required for growth. (A) WT and strains lacking single or (B) multiple TA loci grow similarly in rich media. (C) Induction of the toxins *relE*, *relE-3*, *sehA*, shpAB* or *vapC* arrested or delayed *S*. Typhimurium growth in broth. (D) Expression of corresponding antitoxins with toxins reduced the growth delay. For C and D, overnight cultures were diluted and grown at 37°C for 2 hours, exposed to L-arabinose (0.2%) at Time 0, and monitored by absorbance at OD_600_. Strains carrying plasmid pBAD_33_ or pBAD_24_ were used for comparison; only growth of the strain containing pBAD_33_ is shown because the two vector containing strains grew similarly. Mean of > 3 independent experiments are shown; error bars denote SEM.

**Fig 4:**
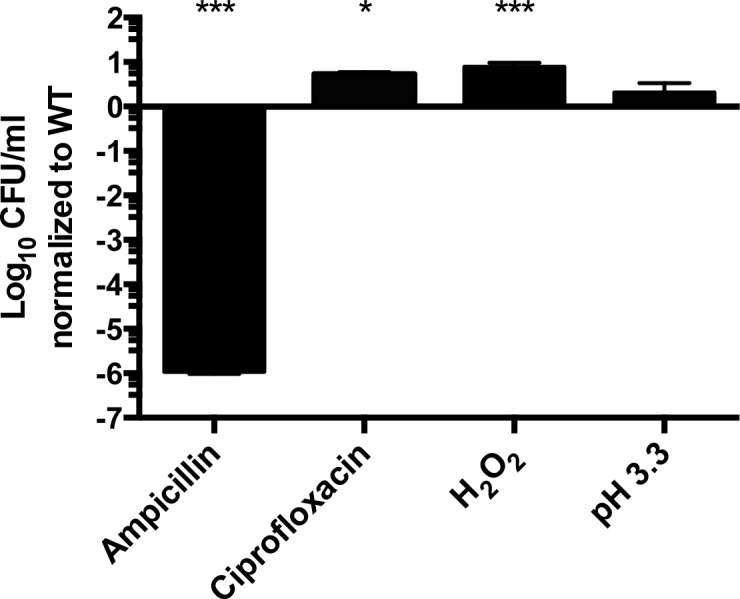
TA loci alter tolerance to antibiotics and hydrogen peroxide. WT or a strain lacking all six TAs were grown in M9-Glucose until late log phase and then challenged for 2 hours with ampicillin (100 μg/mL), ciprofloxacin (0.2 μg/mL), hydrogen peroxide (1.25 mM), or pH 3.3. Results are shown as log_10_ survival after 2 hours of stress relative to wild type. Mean of > 3 independent experiments performed in triplicate are shown; error bars denote SEM. One way ANOVA compared to WT *P <* 0.05 (*), *P <* 0.0001 (***).

The toxins of TA systems are thought to be dispensable for growth in the absence of stress [16,45]. We therefore constructed strains with open reading frame deletions of each of the five *relBE* family members or *vapBC*, and also strains lacking two, three, four, five or all six loci (Table 1). Each of these mutant strains grew as well as wild type in rich and minimal media (*P >* 0.05, Fig 3A and 3B), indicating the five conserved RelE family members and VapC are dispensable for bacterial growth under non-stress conditions. To establish whether the presence of the six TA loci studied is necessary for tolerance, we examined strains lacking these TAs for survival in response to stress. Total colony forming units (CFU) recovered after exposure to ampicillin revealed greatly reduced survival in the absence of all 6 TAs. However, exposure to ciprofloxacin or hydrogen peroxide resulted in slightly increased tolerance compared to a wild-type strain (Fig 4). Other groups have observed that inactivation of multiple TAs results in no change in tolerance or in decreased tolerance [19,25,29]. Overall, the data suggest that genetic removal of TA loci has the potential to increase or decrease tolerance under different kinds of stress.

### *relE*, *relE-3* or *vapC* induction delays or arrests *S*. Typhimurium growth in broth

A hallmark of a toxin is that induced expression of the toxin is sufficient to arrest bacterial growth [46]. Therefore, we cloned each putative toxin and TA pair onto a low copy plasmid under the control of an arabinose-inducible promoter [47]. Control plasmids contained the confirmed toxins *sehA* [24] or shpAB*, which contains the truncated unstable *shpB* antitoxin (referred to here after as shpAB*). Slattery *et al*. showed that shpAB* expression increases tolerance to antibiotics, and that expression of *shpA* alone is lethal [26]). In the absence of arabinose, each strain grew as well as wild type (*P >* 0.05). However, addition of arabinose to the media arrested growth in strains harboring plasmids containing control toxins, *relE*, *relE-3*, or *vapC* (Fig 3C). In contrast, growth was similar in strains with the empty vector or with plasmids expressing *relE-5*, consistent with observations that toxin overexpression does not always affect growth [24]. As anticipated, arabinose induction of putative antitoxins with cognate toxins reduced the effect of the toxin and delayed, instead of arrested, growth (Fig 3D). These experiments show that exogenous expression of some toxin family members arrests or delays bacterial growth.

### The effect of toxin induction on tolerance is variable

**Fig 5:**
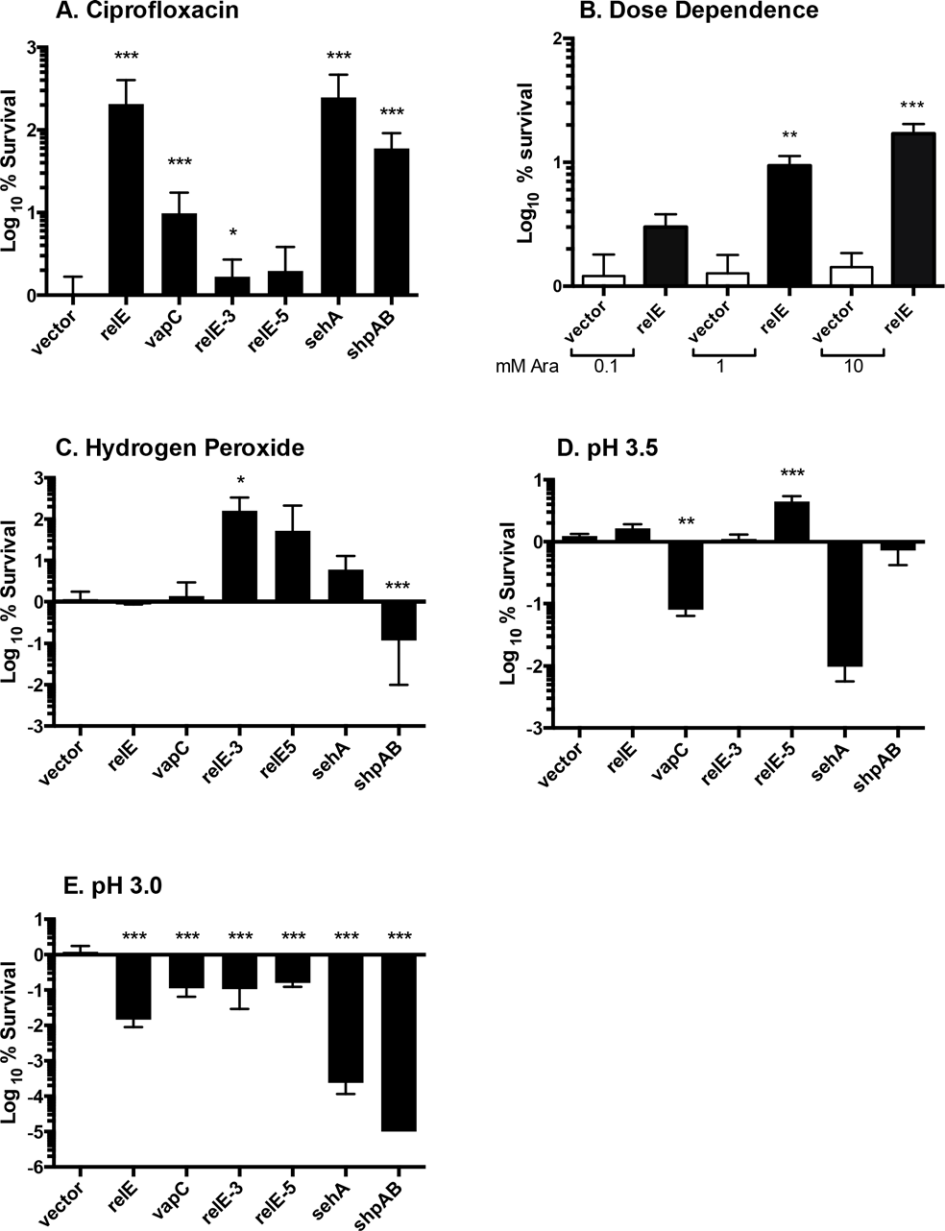
Induction of *relE* increases tolerance to ciprofloxacin, *relE-3* to hydrogen peroxide, and *relE-5* to *pH 3*.*5*. (A) Cells carrying plasmids containing toxins as indicated were grown in M9-Glucose until late log phase and then exposed to 0.2% (12 mM) L-arabinose for 2 hours, followed by 2 hour challenge with ciprofloxacin, 0.2 μg/ml. (B) Dose response. Cells bearing *prelE* were induced with 0.1–10 mM L-arabinose followed by challenge with ciprofloxacin (0.2 μg/mL) for 2 hours. (C—E) As in (A), but challenged with hydrogen peroxide (1.25 mM), pH 3.5, or pH 3.0. Survival was monitored by plating for CFU and results are shown as log_10_ survival after stress, normalized to vector control. Mean of > 4 independent experiments performed in triplicate are shown; error bars denote SEM. A Student's t-test was used to calculate significance comparing each toxin to vector; *P*<0.05 was considered significant. *P <* 0.0001 (***), *P <* 0.001 (**), *P* <0.05 (*).

Exogenous expression of the toxin RelE in *E*. *coli* and the toxin-truncated-antitoxin ShpAB* within *S* Typhimurium increases the fraction of cells tolerant to antibiotic stress [26,48]. To establish whether expression of the conserved five RelE toxins or VapC also increases tolerance to antibiotics, we grew strains harboring inducible toxins to mid-log phase and challenged them with ciprofloxacin (Fig 5A). Induction of three *relE* family members or *vapC* significantly increased tolerance compared to vector control. As expected, induction of the *relBE* TA operon instead of toxin alone did not increase tolerance to ciprofloxacin (*P >* 0.05, data not shown). We titrated arabinose to establish dose dependence using *relE* and indeed, tolerance to ciprofloxacin was a function of arabinose concentration (Fig 5B). We next examined tolerance to hydrogen peroxide and low pH. Only *relE-3* demonstrated increased tolerance in response to hydrogen peroxide, and *relE-5* to pH 3.5 (Fig 5C and 5D). Induction of *vapC* at pH 3.5 and all toxins tested at pH 3.0 surprisingly decreased bacterial survival, perhaps implying a need to tightly repress certain toxins under particular conditions (Fig 5E). These studies suggest that expression of toxins from the VapC or RelE families can positively or negatively affect tolerance.

### *relE-5*, *sehA*, *relE* and *vapC* are induced by *S*. Typhimurium in macrophages

**Fig 6:**
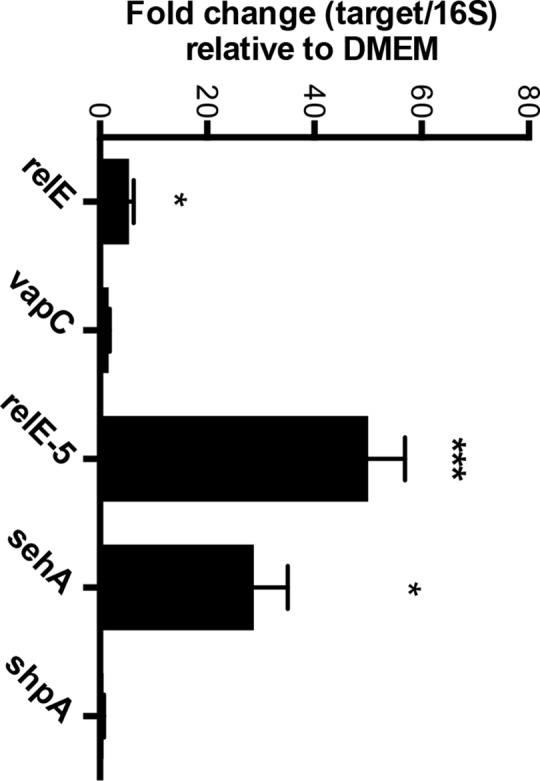
Increased expression of some toxins in macrophages. mRNA was isolated from *Salmonella*-infected bone marrow derived macrophages pre-treated with IFNγ. The level of toxin mRNA was monitored by semi-quantitative RT-PCR and fold change relative to 16S RNA is shown. mRNA from *relE-4* was not detected. Mean of > 3 independent experiments performed in triplicate are shown; error bars denote SEM. Two tailed Student’s *t-test* comparing macrophage expression to expression in DMEM, *P <* 0.05 (*), *P <* 0.0001 (***). Growth of *S*. Typhimurium in DMEM and LB were equivalent.

To establish systemic infections, *S*. *enterica* colonizes and disseminates within macrophages. Therefore, we determined whether the six TA toxins conserved across *S*. Typhimurium that cause systemic infection are expressed in macrophages. Total CFU recovered from macrophages infected for 18 hours with a strain lacking all six TA loci was indistinguishable from wild type (data not shown). We next determined whether bacterial uptake by macrophages induces expression of *relE* and *vapC* family members. We performed semi-quantitative RT-PCR on RNA isolated from primary mouse macrophages. Bacteria grown in tissue culture medium served as a control [49,50]. Two hours after infection, the fold change in bacterial gene expression of three of the toxins significantly increased as compared to controls (Fig 6). *sehA* was induced 28-fold, consistent with expression of this gene in the mesenteric lymph nodes of mice [24]. The *relE-5* gene was induced 50-fold, *relE*, five-fold, and *vapC*, three-fold. In summary, RT-PCR reveals that some but not all of the toxins examined appear to be expressed during infection of macrophages.

## Discussion

Persister bacteria play a major role in recalcitrant infections and are an important factor in the failure of antibiotic therapies (5). In this study we show that exposure of *S*. Typhimurium to antibiotics and some conditions that mimic host microenvironments reveal not simply tolerance or resistant mutations, but a persister state, as defined by the observation of similar fractions of tolerant bacteria upon re-exposure to stress [3]. Oxidative stress and low pH exposure revealed a subpopulation of bacteria tolerant to these stresses. Under acidic conditions, we did not observe the significant bacterial killing that is necessary to reveal persisters until the pH was as low as 3.5. However, pre-exposure of bacteria to pH 4.5 for only 30 minutes is sufficient to increase the percentage of bacteria tolerant to antibiotics [25], indicating that moderately stressful conditions have the potential to pre-dispose bacteria to tolerance to a second stress. These data collectively suggest that single stressors consistent with the host environment may select for a small fraction of bacteria that are tolerant, and that combinations or sequential addition of stressors may allow bacteria to adopt a phenotypic persister state.

Toxin-antitoxin systems contribute to the establishment of a persister-like state by reversibly blocking cellular processes necessary for growth or by causing damage [45]. Of the six TA loci conserved across *Salmonella enterica* subspecies that cause systemic infections in mammals and birds, exogenous expression of *vapC* or *sehA* resulted in complete growth arrest. In addition, ectopic expression of three RelE family member toxins enhanced tolerance to ciprofloxacin (*relE*), hydrogen peroxide (*relE-3*) or low pH (*relE-5*). Similarly, ectopic expressions of select toxins in *Burkholderia cenocepacia* inhibit growth, whereas others confer tolerance: induction of a *B*. *cenocepacia* RelE or VapC increased biofilm formation [51]. Induced expression of PasT in uropathogenic *E coli* increased tolerance to nitrosative and oxidative stress and was required for survival in the kidney but not in the bladder [52]. These observations support the hypothesis that different toxins have the capacity to protect bacteria from different stressors [19,27]. However, we also found that S. Typhimurium expresses certain toxins in macrophages, but not the same toxins that conferred tolerance to the stressors we tested. This result may reflect that some toxins are important under simpler conditions and others in more complex environments such as the macrophage SCV. Alternatively, bacterial toxin expression in macrophages may be highly heterogeneous and therefore not amenable to population assays, such as RT-PCR or plating for CFU.

While exogenous expression demonstrates that toxins are sufficient to confer tolerance under some circumstances, none of the six toxin-antitoxin loci examined were necessary to protect bacteria from antibiotic, low pH, or hydrogen peroxide stress. This may reflect functional redundancy of TA systems, as demonstrated in *E*. *coli* [29]. Functional redundancy of TA systems was also observed in *S*. Typhimurium upon deletion of individual or multiple TAs, which reduced bacterial persister formation in macrophages by as much as 90% after 30 minutes. However, by 18 hours, the proportion of non-replicating bacteria is only modestly reduced (< 30%) [25]. These data suggest that while individual TA systems contribute at early stages of infection to a persister phenotype, their contributions are masked within 18 hours by redundant genes, either TAs or other loci. In other words, in the absence of certain TA systems, other genes may compensate in a proportion of the population.

Along these lines, we unexpectedly found that a strain lacking all five RelBE TA loci as well as the VapBC locus had increased tolerance upon exposure to hydrogen peroxide and reduced tolerance to ampicillin. Previously reported deletion of toxins had no detectable phenotype or reduced bacterial survival under some conditions. For instance, deletion of the gene encoding the toxin VapC*2* reduces *S*. Typhimurium survival in HeLa cells, whereas the toxin T4 is needed in fibroblasts [19]. We speculate that the changes in tolerance observed in broth upon deletion of the six TA loci reflects complex gene regulation, for instance, by stochastic degradation of antitoxins [10]. Future progress in this realm will depend upon single cell approaches.

## Supporting Information

S1 Table Minimal data set. Data for figures is supplied.
(XLSX)
